# A loss of function mutation in SOCS2 results in increased inflammatory response of macrophages to TLR ligands and *Staphylococcus aureus*

**DOI:** 10.3389/fimmu.2024.1397330

**Published:** 2024-08-09

**Authors:** Laurence Guzylack-Piriou, Blandine Gausseres, Christian Tasca, Chervin Hassel, Guillaume Tabouret, Gilles Foucras

**Affiliations:** IHAP, Université de Toulouse, INRAE, ENVT, Toulouse, France

**Keywords:** SOCS2, R96C mutation, inflammatory response, macrophages, mice

## Abstract

**Introduction:**

The role of suppressor of cytokine signaling (SOCS)2 in anti-infective bacterial immunity has been poorly investigated compared to other members of the SOCS family.

**Methods:**

We characterized the previously identified loss of function R96C point mutation of SOCS2 using a genome-edited mouse model that resumes the phenotype of Socs2 knockout mice. The response of macrophages to TLR-ligands and *Staphylococcus aureus* was examined.

**Results and discussion:**

Conversely to previously published data using human monocyte-derived macrophages, the stimulation of bone-marrow-derived macrophages with various TLR ligands did not show any difference according to the SOCS2 variant. Upregulation of IL-6 and TNF-α pro-inflammatory cytokines production was only seen when the SOCS2 expression was promoted by the culture of macrophages in the presence of GM-CSF. Furthermore, we showed that the SOCS2 point mutation is associated with heightened STAT5 phosphorylation in a short time frame upon GM-CSF incubation. In mice, recruitment of neutrophil and F4/80^int^ Ly6C^+^ inflammatory macrophage, as well as IFN-γ and IL-10 concentrations, are significantly increased upon *S. aureus* peritoneal infection. Altogether, these data support the idea that by lowering the pro-inflammatory environment, SOCS2 favors better control of bacterial burden during a systemic infection caused by *S. aureus*.

## Introduction

In recent years, it has become increasingly evident that suppressor of cytokine signaling (SOCS) proteins have important roles in the maintenance of homeostasis and resolution of inflammatory processes.

Suppressors of cytokine signaling (SOCS) proteins negatively regulate JAK-STAT and consist of eight members: SOCS1-7 and cytokine-inducible SH2-containing protein (CIS) (1). The STAT family of transcription factors plays a critical role in regulating physiological responses to cytokine stimulation. Members of the STAT family bind tyrosine-phosphorylated cytokine receptors through their SH2 domains. Once bound to the receptor, STATs are phosphorylated by JAKs, which causes them to dissociate from the receptor and form homo- or hetero-dimers. STAT dimers then translocate to the nucleus, where they interact with specific DNA elements in the promoters of target genes and thus regulate transcription (2).

Thus, SOCS proteins are likely to be involved in the differentiation of cells involved in innate and adaptive immunity, helping to shape the inflammatory response (3). Importantly, SOCS proteins appear to modulate CD4^+^ T-cell polarization (4). Moreover, a peptidomimetic of SOCS1 has been shown as valuable therapeutics in several disorders (5).

This is exemplified by the regulation of the differentiation of Th2 (T helper 2) cells by SOCS3 and SOCS2 (4, 6), whereas Th17 differentiation is regulated by SOCS1 and SOCS3 (7, 8). Moreover, Knosp et al. showed a dual role for SOCS2 in both Th2 and Foxp3^+^ iTreg generation (9). The proteasome dependent pathway of SOCS2 is triggered by lipoxins to modulate innate immune signaling (10). Indeed, lipoxins can induce SOCS2-dependent ubiquitinylation and proteasomal degradation of TRAF6, hindering pro-inflammatory cytokine expression by dendritic cells. SOCS2 is also a well-established negative regulator of growth hormone signaling via the JAK2/STAT5 pathway (11, 12), but its role is less well-established in immune functions.

The two main functional domains of the SOCS2 protein are the family-related C-terminal SOCS box and the SH2 region. The SOCS box mediates the assembly of elongin B/C-cullin complexes to facilitate ubiquitination processes, leading to proteasomal degradation of the ligand (13). Most importantly, the SH2 domain interacts with the substrate by recognizing phosphorylated tyrosine residues in the intracellular domain of several receptors (14), interfering with the downstream signaling due to competition with signaling molecules or by promoting receptor degradation with a lower cell surface expression. The SOCS2-SH2 domain has also been reported to directly interact with JAK2 (15).

Several studies using Socs2^KO^ mice have highlighted the contribution of SOCS2 in regulating immune cell function in specific inflammatory or infectious contexts (16). SOCS2 deficiency induces hyper-responsiveness of dendritic cells (DCs) to microbial stimuli (17) and is related to an unbalanced inflammatory response during *Toxoplasma gondii*, *Trypanosoma cruzi*, and *Plasmodium berghei* infections (18). The action of acetylsalicylic acid, an anti-inflammatory drug, was shown to be dependent on SOCS2, confirming its role in both the immune response to infection and the regulation of inflammatory processes (17).

A recent study has identified a point mutation in SOCS2 about an increased inflammatory response (19), and a higher prevalence of infections by *Staphyloccoci* (20). This mutation changes the arginine at position 96, located in the SH2 domain, into a cysteine (p.R96C), leading to a diminished binding affinity for phosphorylated ligands, further demonstrating the importance of SH2:pTyr binding to the functions of SOCS2 (1, 15).

The role of SOCS2 has been little investigated during bacterial infection. Few reports have noted its involvement in the regulation of bacteria-associated inflammation, as well as lipopolysaccharide (LPS) signaling (21). Some data suggest that SOCS2 may target and mediate proteasome-dependent degradation of other SOCS proteins, like SOCS1 and SOCS3 (22, 23). The expression of SOCS2 was shown to increase in macrophages infected with mycobacteria, and *SOCS2*-deficient mice exhibit higher sensitivity to the inflammation induced by *Mycobacterium bovis* (24). Nonetheless, most studies have concluded that the activity of *SOCS2* is limited and redundant, in contrast to our field observations of higher predisposition to staphylococcal infections in animals carrying the SOCS2 variant in a homozygote state (19).

Identification of a loss-of-function (LOF) mutation in SOCS2 provides a good opportunity to study the role of SOCS2 in the context of infection, using a more physiological model than with knockout mice. We developed a genome-edited mouse model to express the R96C SOCS2 variant to characterize the consequence of SOCS2 invalidation during the inflammatory response. Here, we show that SOCS2 plays a more important role in the regulation of the response to bacterial infection and inflammation than initially thought.

## Materials and methods

### Mouse strains

The study was carried out in compliance with the ARRIVE guidelines (http://nc3rs.org.uk/arrive-guidelines). The R96C point mutation was introduced into the C57Bl/6 mouse genome by homologous recombination using CRISPR/Cas9 technology. One single nucleotide was replaced by homologous recombination in pronuclear-stage zygotes, as previously described (25). The C57Bl/6 background was chosen to facilitate the production of further recombinant mice (gene reporter or knockout strains) of interest to elucidate the mechanisms altered by the point mutation. Eight- to 10-week-old female or male SOCS2^KI^ mice or their littermates WT mice were bred and housed in a specific pathogen-free facility (INSERM US 006 – CREFRE). Body growth was determined for female and male WT and SOCS2^KI^ mice. Experiments were performed in an accredited research animal facility of the UMR IHAP, ENVT, Toulouse, France. Mice were handled and cared for according to the ethical guidelines of our institution (APAFIS#22936-2019112515186332) following the Guide for the Care and Use of Laboratory Animals (National Research Council, 1996) and in compliance with European directive 2010/63/UE under the supervision of authorized investigators. Mice were euthanized by cervical dislocation and all efforts were made to minimize the pain and distress of the animals.

### Computed tomography imaging of living mice

Anesthetized (Isoflurane, Virbac) 10-week-old male mice (n=6) were scanned using a small animal computed tomography (CT) system (NanoScan PET/CT Mediso). The nanoScan CT has a rotating gantry and the X-ray source and detector rotate around the object. The scanning protocol was 35 kVp, 800 µA, 450 ms integration time, 720 projections per 360°, scan duration of 5’33”. The reconstructed voxel size was 125 x 125 x 125 µm. From the acquired data, the length of the humerus, radius, ulna, femur, and tibia and the length and width of the skull were bilaterally determined. To ensure the reproducibility of the measurements, precise anatomical landmarks were defined beforehand.

### Preparation of *S. aureus* and challenging of the mice

*Staphylococcus aureus subsp. aureus (ATCC^®^ 49525™)* XEN36 (bioluminescent bacterial strain) or the HG001 strain, a GFP-expressing mutant (26), were grown overnight in TSB at 37°C with orbital shaking (200 rpm). The culture was further diluted 1:100 in TSB and grown to the mid-log phase (O.D. 600 nm _1). Bacterial cells were pelleted (2800 x g, 10 min, 4°C), washed twice in PBS, and resuspended in physiological serum. The concentration of the bacteria was estimated by measuring the absorbance at 600 nm (OD600 = 0.8 for 2×10^8^ CFU/mL) and confirmed by serial dilution in PBS Tween 20 (0.05%) and plating on LB-Agar for determination of the number of colony-forming units (CFU). CFUs were determined after 24 h of incubation at 37°C. Bacteria were freshly prepared before each experiment and adjusted to the desired concentration. The XEN36 strain was killed by heating for 1 h at 65°C. *S. aureus* inactivation was confirmed by LB culture. Male mice were infected intraperitoneally (i.p.). with *S. aureus*. at 10^8^ CFU/mouse (100 ul/mouse).

### Peritoneal cell collection and spleen and lymph node cell isolation

The peritoneal cavity was washed by injecting 3 mL PBS. After recovery, peritoneal exudates were centrifuged (300 x g, 5 min) and the supernatants were stored at -80°C for further analysis. PerCs were harvested for cell phenotyping by flow cytometry. The spleens and lymph nodes (LNs) were removed and the cells were isolated through 70 and 40 μm cell strainers, respectively, to obtain single-cell suspensions in PBS.

### Flow cytometry analysis

The number of cells obtained from the peritoneal exudates, spleens, and LNs was determined using a flow cytometry absolute counting system (MACSQuant Analyzer, Miltenyi Biotec, Germany). Cells (1-2 x 10^6^) were incubated in HBSS, 0.5% BSA, 10 mM Hepes containing mouse FcR Blocking Reagent (Miltenyi Biotec, Germany) following the manufacturer’s instructions. Cell viability was assessed using Viobility 488/520 Fixable Dye (Miltenyi Biotec, Germany). Antibodies were incubated at 4°C for 30 min in the dark. The antibodies used for flow cytometry are listed in **Supplementary Table 1**. Flow cytometry was used to measure efferocytosis by quantifying the number of F4/80^int^ Ly6G^+^ macrophages in the peritoneal cavity from WT or SOCS2^KI^ mice after *S. aureus* infection. The acquisition was performed using a MACSQuant (Miltenyi Biotec, Germany) flow cytometer with MACS Quantify software. Flow cytometry data were analyzed using FlowJo (Tree Star, USA) software.

### Preparation of mouse bone marrow-derived macrophages

Femurs and tibias from male WT or SOCS2^KI^ mice were cut at both ends and the bone marrow was flushed out with PBS with a syringe mounted with a 26-gauge needle. The cells were collected and cultured in X-Vivo (BE02-060F, Ozyme, France) supplemented with 50 ng/mL M-CSF (Peprotech, France) at a density of 2 x10^5^ cells/mL. Cells were incubated at 37°C in 5% CO_2_ and fresh medium was added on days 3 and 5 of culture. On day 7, the BMMs were harvested, counted, and primed for 24 h with either GM-CSF (Biolegend, France), IFN-γ (Peprotech, France), or IL-10 (Peprotech, France) at 3, 10, or 30 ng/ml. The TLR ligands FLS1 (Invivogen, France) and CRX (Invivogen, France) at various concentrations (3, 10, 30, or 100 nM) or heat-killed *S. aureus* (HKSA) (Invivogen, France) or heat-killed *E. coli* (HKEB) (Invivogen, France) at various MOIs (3, 10, 30, or 100) were added to the cell cultures, and the cultures were incubated for an additional 24 h. We used the mycoplasma lipoprotein FSL-1, recognized by the TLR2/TLR6 heterodimer, HKSA, recognized mainly by TLR2, CRX-527, a highly specific TLR4 agonist and an LPS-like molecule, and HKEB recognized by TLR2 and TLR4. Cell supernatants were then harvested for cytokine detection. BMMs were pulsed 24 h with GM-CSF at 10 ng/ml to induce SOCS2 expression followed by a 3 h chase. Then, GM-CSF (10 ng/ml) was added *de novo* to cells followed by incubation for 10, 30, 60, or 120 min. Cells were harvested to analyze SOCS2 and pSTAT5/total STAT5 expression by western blotting.

### Measurement of cytokine/chemokine production

Cytokines in peritoneal exudates were quantified using a customized multiplex assay kit containing IL-1β, IL1-α, IL-10, IL-6, IFN-γ, IL-12, IL-17, CXCL1, CXCL10, CCL2, CCL3, CCL4, and TNF-α (Tumor necrosis factor) (Milliplex-MAP, Merck Millipore, France) and a xMAP instrument (MAGPIX, Luminex). Individual cytokine detection kits were also used to quantify mouse IL-6 and TNF-α (Bio-techne, France) and GM-CSF (Biolegend, San Diego, USA).

### Western-blot analysis for SOCS2 protein or related nuclear transcriptional factors

After stimulation, BMMs were washed once with cold PBS. Total protein was extracted with radio immune precipitation assay (RIPA) buffer containing phenylmethylsulphonyl fluoride (PMSF) (Roche, Switzerland) and a protease inhibitor cocktail (Roche) before storage at -80°C. Protein concentrations were determined using the BCA method. Cell lysates (30 µg per lane) were subjected to SDS polyacrylamide gel electrophoresis and then transferred to polyvinylidene difluoride membranes for western-blot analysis. After blocking with 5% fat-free milk dissolved in TBS-T (Tris-buffered solution with Tween 20) for 1 h at room temperature, membranes were incubated overnight with antibodies raised against pSTAT5, total STAT5, and SOCS2 (Cell Signaling Technology, Beverly, MA) according to the manufacturer’s instructions (**Supplementary Table 2**). The binding of these primary antibodies was visualized using goat anti-rabbit/anti-mouse immunoglobulin coupled with horseradish peroxidase (Jackson ImmunoResearch). The measurement of β-actin served as a loading control.

### Phagocytosis assay

To assess phagocytosis, BMMs were first primed with either GM-CSF (Biolegend, France), IFN-γ (Peprotech, France), or IL-10 (Peprotech, France) for 24 h and then infected at a MOI of bacteria to macrophages of 10:1 with *S. aureus* HG001-GFP for 1 h at 37°C or 4°C as a control. Each well was washed three times and extra-cellular fluorescence quenched by adding 0.2 uM syringe-filtrated trypan blue (Sigma). Cell viability was determined using 7-AAD dye (BioLegend). The amount of live KI and WT BMMs with internal (BT-) engulfed bacteria (GPF+) was measured by flow cytometry (MACSQuant, Miltenyi Biotech, Germany).

### *In vivo* bioimaging

Male mice were infected i.p. with bioluminescent *S. aureus* XEN36 at 10^8^ CFU/mouse (100 ul/mouse). A neutrophil elastase solution (680 FAST, Perkin Elmer) was injected intravenously (4 nmol/100 ul/mouse) 3 h later. The animals were imaged by bioluminescence and fluorescence 24 h after *S. aureus* infection.

### Quantification and statistical analysis

Prism v. 8.0.1 (GraphPad Software, San Diego, CA, USA) was used for all analyses. Significant differences were analyzed using the t-test or multiple t-tests of ANOVA. P<0.05 was considered statistically significant. Statistical computing was carried out and some of the graphics were generated using R software. Graphics were designed using the ggplot2 R package. Heatmaps were computed after the transformation of the cytokine concentrations into Z-scores and generated using the Pheatmap R package (Kolde Raivo). PLSDA was carried out using the MixOmics package (27).

## Results

### The mouse model carrying the loss of function R96C point mutation in SOCS2 resumes the *Socs2* knockout phenotype

**Figure 1:**
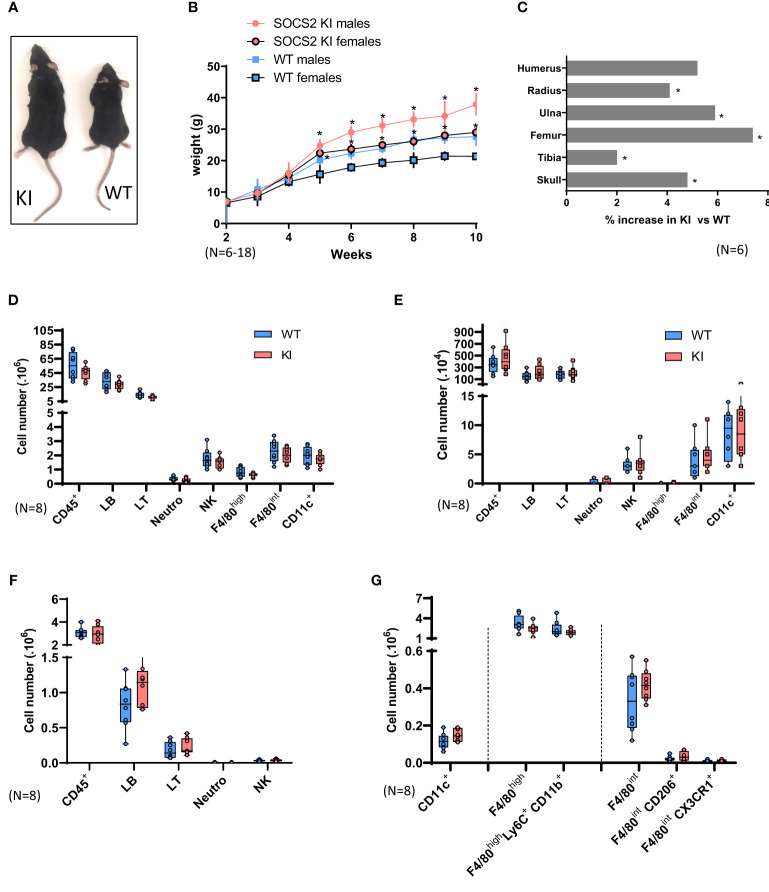
Characterization of SOCS2^R96C^ KI Mice. **(A)** Increase in the size of a typical two-month-old SOCS2^KI^ male (left) relative to an age- and sex-matched wildtype animal (right). **(B)** Growth curves for male and female SOCS2^KI^ (circles) and WT (squares) mice. The body weight of the mice, at weekly intervals, is shown. Each point represents the mean ± SD for 6-18 mice. **(C)** Percentage increase in bone length in SOCS2^KI^ mice (right and left) (N=6). Flow cytometry analysis of the immune landscape of single-cell suspensions from the **(D)** spleen, **(E)** lymph nodes, and **(F)** peritoneal cavity of two-month-old male SOCS2^KI^ or WT mice. (N=8). **(G)** Flow cytometry analysis of macrophages/dendritic cell subsets from the peritoneal cavity of two-month-old male SOCS2^KI^ or WT mice, (N=8). Statistical analysis was performed using the multiple t-test and significant p values are indicated. *P<0.05 vs. WT. SOCS, suppressor of cytokine signaling; WT, wildtype; KI, SOCS2^KI^ mice.

For further investigating the R96C SOCS2 mutation *in vivo*, we used CRISPR/Cas9 gene editing to generate a new C57BL/6 mouse strain bearing the Arg96 to Cys mutation (SOCS2^KI^ mice). SOCS2^KI^ mice were indistinguishable from wildtype (WT) mice until weaning at three weeks of age, but they subsequently grew more rapidly (**Figure 1A**), and achieved higher body weight (average of 40%) by six weeks of age, in both male and female mice (**Figure 1B**). CT imaging of skeletons from 10-week old males revealed that the length of most long bones and skull was significantly increased (**Figure 1C**), showing that the gigantism phenotype of *SOCS2^-/-^* mice is resumed in the R96C knock-in model. In line with our previous observation, the R96C mutation is associated with a loss of SOCS2 functions. We next characterized the immune-cell composition of various immune organs and compartments, such as the spleen, LNs, and peritoneal cavity of SOCS2^KI^ mice relative to those of WT mice (**Supplementary Figure 1**). No significant difference was observed in the immune-cell composition of the spleen (**Figure 1D**), the LNs (**Figure 1E**), or the peritoneal cavity (**Figure 1F**). However, the cell number per unit of body weight was significantly lower in SOCS2^KI^ mice, as shown for the spleen (**Supplementary Figure 2**).

Due to the previously reported important role of SOCS2 in these cell types, we next analyzed macrophage and DC populations in the peritoneal cavity and spleen of SOCS2^KI^ mice in comparison with WT mice (**Figure 1G**; **Supplementary Figure 3**). At the steady state, there were no significant differences in the cell composition, and the cell numbers of various macrophages or DCs types were similar in the two genotypes.

### SOCS2 is not induced by TLR ligation in M-CSF-derived macrophages and is only detectable after incubation with GM-CSF

In mice, bone-marrow macrophages differentiated using M-CSF as a growth factor are a frequently used and convenient model to study signaling and functions in macrophages (28). After M-CSF culture of bone marrow stem cells, no difference in the phenotype is noticeable between WT and SOCS2^KI^ mice. Both BMM cultures showed a homogeneous population of F4/80^+^ cells, with similar levels of CD11b and MHCII molecule expression (**Supplementary Figure 4**).

**Figure 2:**
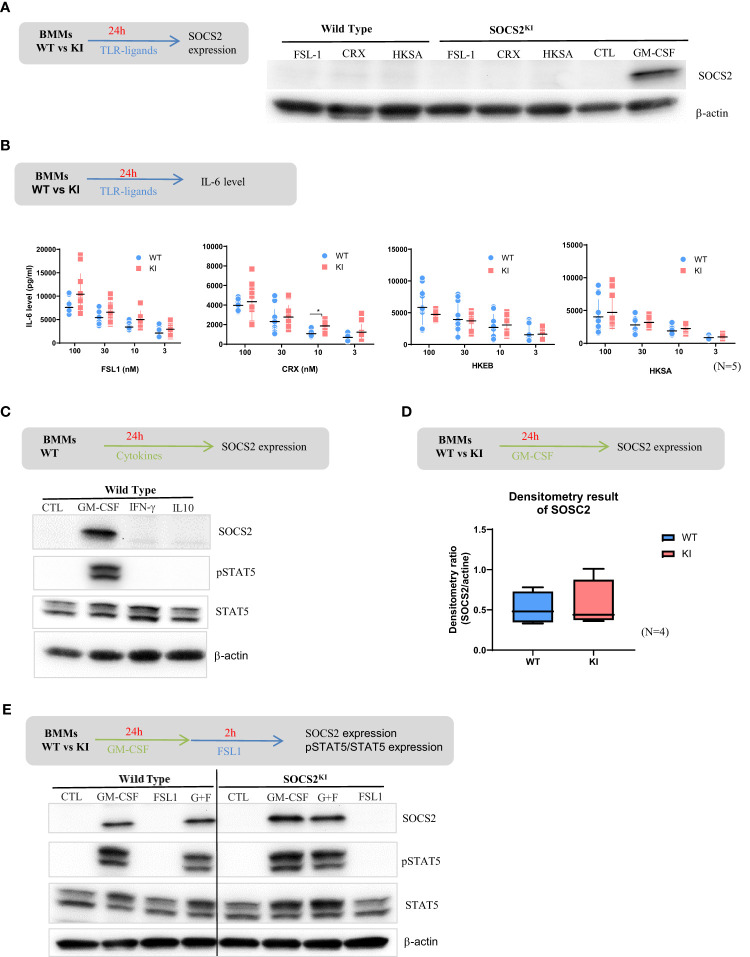
Response of BMMs from SOCS2^KI^ or WT mice to TLR ligands or cytokines. BMMs from SOCS2^KI^ or WT mice were stimulated for 24 h with FSL1, CRX, HKSA, or HKEB TLR ligands at 100 nM. **(A)** SOCS2 expression in cells was determined by western blotting. **(B)** BMMs from SOCS2^KI^ or WT mice were stimulated for 24 h with 3 to 100 nM of FSL1, CRX, HKSA, or HKEB TLR ligands. IL-6 concentrations were measured in cell supernatants. Controls without stimulation were below the detection limit of cytokine assay (Mean ± SD, N=5) *P<0.05 vs. WT, by multiple group comparison of ANOVA. **(C)** BMMs from SOCS2^KI^ or WT mice were cultured with the cytokines GM-CSF, IFN-γ, or IL-10 at 30 ng/ml for 24 h, and SOCS2, pSTAT5, and STAT5 expression was evaluated by western blotting. **(D)** Density results of SOCS2 expression/actin. **(E)** BMMs from SOCS2^KI^ or WT mice were cultured with the cytokines GM-CSF at 30 ng/ml for 24 h and stimulated 2h with FSL1 ligands at 100 nM. SOCS2, pSTAT5, and STAT5 expression in cells, was determined by western blotting. Blots are representative of three independent experiments.

Evidence has now accumulated indicating that SOCS1, SOCS3, and CIS are induced after TLR engagement in macrophages and DCs, and this contributes to avoid overshooting TLR stimulation (29). Similarly, it was previously shown (30) that various TLR ligands can induce *Socs2* gene expression in human DCs or thioglycolate-induced mouse peritoneal macrophages (31). We thus investigated SOCS2 protein expression after TLR stimulation by western blot in WT and SOCS2^KI^ M-CSF-derived macrophages. To our surprise, neither TLR-2 (FSL1, HKSA) nor TLR-4 (CRX) ligands induced SOCS2 expression in BMMs from both WT and SOCS2^KI^ mice after 24 h of culture (**Figure 2A**) in contrast to previous reports using GM-CSF as a differentiating factor. We also investigated the direct effect of TLR ligands on pro-inflammatory cytokine production in these conditions. There was no or few difference in the secretion of IL-6 in the cell culture supernatants of BMMs from WT and SOCS2^KI^ mice stimulated with TLR ligands like FSL1 or CRX, or heat-killed bacteria like HKSA or HKEB, regardless of the concentration we used (**Figure 2B**).

However, it is worth noting that FSL1 stimulation induced higher production of GM-CSF by the BMMs from SOCS2^KI^ than WT mice (**Supplementary Figure 5**).

SOCS2 is a well-known feedback inhibitor of the JAK-STAT signaling, and STAT5-inducing cytokines, such as GM-CSF promote its expression (32, 33), whereas IFN-γ via STAT-1 and IL-10 via STAT-3 induces SOCS1 (34) and SOCS3, respectively (as reviewed in 35). Indeed, SOCS2 was found expressed in BMMs from WT mice after 24h culture with GM-CSF, but not with IFN-γ or IL-10 (**Figure 2C**). After GM-CSF culture, SOCS2 was also expressed by BMMs from SOCS2^KI^ mice at comparable levels as indicated by densitometry results on western blot data (**Figure 2D**). We then detected STAT5 in WT BMMs whatever the conditions, but phospho-STAT5 was only seen when GM-CSF was present in the culture medium (**Figure 2C**). Further stimulation with FSL-1 did not substantially change STAT5 expression, nor its phosphorylation. No difference in pSTAT5/STAT5 levels were noticed between the two SOCS2 variants, and SOCS2 was equally well detected (**Figure 2E**).

### Phosphorylation of STAT5 is more sustained in SOCS2^KI^ BMMs after a GM-CSF pulse while SOCS2 is expressed

**Figure 3:**
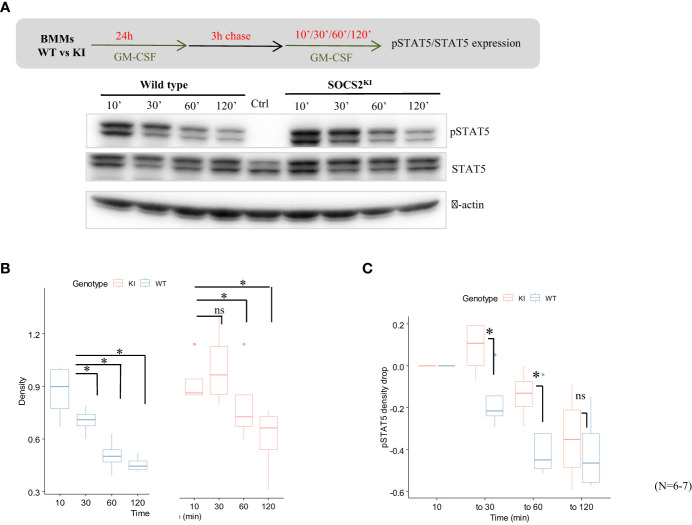
Kinetics of pSTAT5 expression in GM-CSF-cultured BMMs from SOCS2^KI^ or WT mice. BMMs were cultured in the presence of GM-CSF (10 ng/ml) for 24 h followed by a chase of 3 h in a culture medium without cytokines. **(A)** Expression of pSTAT5 was analyzed after the re-addition of GM-CSF at 10 ng/ml for 10 min to 2 h of stimulation by western blotting. **(B)** Density representation of pSTAT5 blots as a function of duration of stimulation with GM-CSF for WT and SOCS2^KI^ BMMs. **(C)** Drop in pSTAT5 density. *P<0.05; ns, not significant, Kruskal Wallis test.

To investigate the possibility of a difference in the JAK-STAT signaling turnover rate, we next studied the kinetics of STAT5 phosphorylation/dephosphorylation following the stimulation with GM-CSF. For that purpose, both cell types were cultured for 24h with GM-CSF to induce SOCS2 expression, which was followed by a 3-h chase to return to the steady state and absence of phospho-STAT5, as shown in the control condition in **Figure 3A**. Phospho-STAT5 levels were monitored from 10 to 120 min after re-introduction of GM-CSF in the BMM medium (**Figure 3A**). Phosphorylation of STAT5 was already present at high levels in both KI and WT cells after 10 min of stimulation. After that, STAT5 phosphorylation decreased rapidly in WT BMMs while it was sustained at high levels in SOCS2^KI^ cells for up to 30 min post-stimulation; a significant difference was still present after one hour of stimulation (**Figure 3B**). Relative to WT, STAT5 dephosphorylation was significantly delayed in SOCS2^KI^ cells (**Figure 3C**), consistent with our working hypothesis of a difference in the regulation of the JAK/STAT pathway when SOCS2 is inactive.

### Pro-inflammatory responses are enhanced in SOCS2^KI^ BMMs about the cytokine context and the presence of GM-CSF

**Figure 4:**
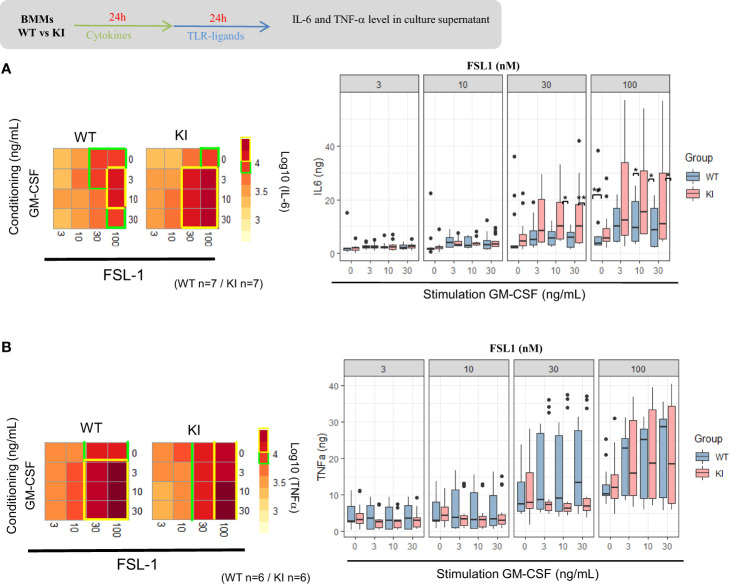
IL-6 and TNF-α response to FSL1 in GM-CSF-conditioning medium. Heatmap representation of IL-6 **(a)** and TNF-α **(b)** concentrations in supernatants of BMMs from SOCS2^KI^ or WT mice (after Z-score transformation) after culture with increasing concentrations of FSL1 (from 3 to 100 nM) and GM-CSF (from 3 to 30 ng/ml). IL-6 **(A)** and TNF-α **(B)** concentrations (pg/mL) in BMM supernatants after culture with increasing concentrations of FLS1 and GM-CSF. Results represent the mean ± SD of 6-7 independent donors. *P<0.05, **P<0.01 vs. WT, by multiple group comparison of ANOVA.

Macrophages secrete both pro- and anti-inflammatory cytokines following initial exposure to microbial-associated molecular patterns (MAMPS). As such, deletion of STAT5 results in increased pro-inflammatory cytokine expression in mouse myeloid cells with PRR (pattern recognition receptor) expression (36), but results are less known if STAT5 signals are increased. We explored the consequences of the SOCS2 LOF mutation on the pro-inflammatory cytokine secretion after activation with TLR2-related ligands, FSL-1 and HKSA. For that, SOCS2^KI^ and WT BMMs were cultured for 24 h with different concentrations of GM-CSF to induce SOCS2, followed by a MAMPS stimulation. Cytokine concentrations (**Supplementary Figure 8**) are depicted as a heatmap according to the FSL-1 (from 3 to 100 nM) and GM-CSF concentrations (from 3 to 30 ng/ml) in main **Figure 4**. Lower concentrations of FSL-1, even in the presence of the lowest concentration of GM-CSF (3 ng/ml) tested here, are sufficient to induce a greater production of IL-6 in SOCS2^KI^ than WT BMMs (**Figure 4A**). Indeed, stimulation with FSL1 (at 30 and 100 nM) resulted in significantly higher production of IL-6 by BMMs from SOCS2^KI^ mice with a 4-fold increase. Interestingly, TNF-α secretion was not affected by the loss of SOCS2 function (**Figure 4B**).

### R96C-SOCS2 modifies the response to IFN-γ and IL-10 cytokines that are known to promote SOCS1 and SOCS3 expression, respectively

Previous studies indicated that SOCS2 may regulate other SOCS proteins, in particular SOCS1 and SOCS3 (22), in a dose-dependent manner (37). Through this mechanism, SOCS2 can indirectly modulate cytokine-induced STAT activation by removing the negative regulation mediated by the two other SOCS proteins that are targeted.

**Figure 5:**
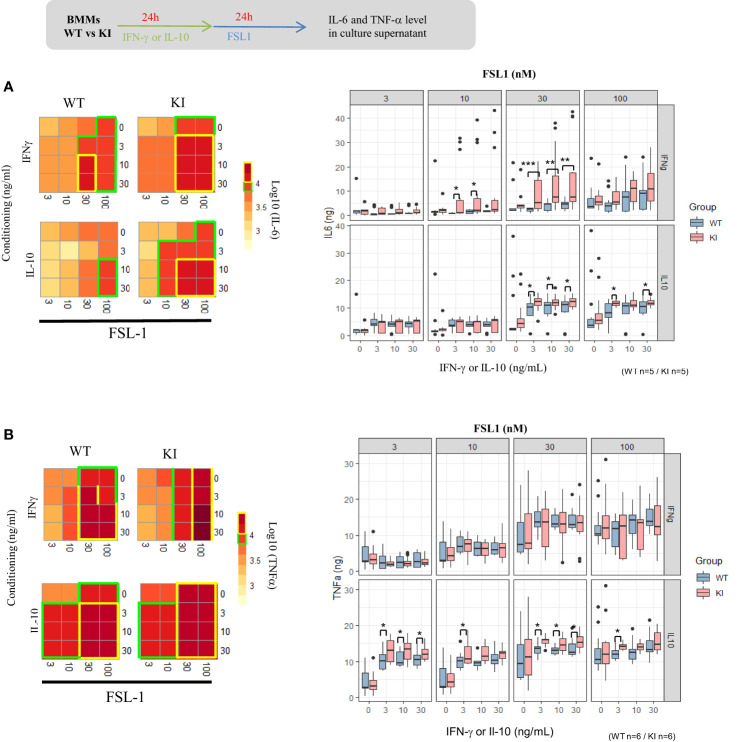
IL-6 and TNF-α response of BMMs to FSL1 in IFN-γ and IL-10 conditioning medium. Heatmap representation of IL-6 **(A)** or TNF-α **(B)** concentrations in supernatants of BMMs from SOCS2^KI^ or WT mice (Z-score transformation) after culture with increasing concentrations of FSL1 (3 to 100 nM) and IFN-γ or IL-10 (3 to 30 ng/ml). Results represent the mean ± SD of 5-6 independent donors. *P<0.05, **P<0.01, ***P<0.001 vs. WT, by multiple group comparison of ANOVA.

We next investigated the possible interaction of SOCS2 on the respective role of IFNγ and IL-10 cytokines known to regulate SOCS1 and SOCS3, respectively. As done with GM-CSF, BMMs were first primed with IFN-γ and IL-10 before stimulation with the FSL-1 ligand. Recognition of FSL-1, a diacylated lipoprotein, is mediated by TLR2 which cooperates with TLR6 through their cytoplasmic domain to induce a signaling cascade leading to AP-1 and NF-κB activation and cytokine production. The cytokine response and phagocytic activity of cytokine-driven macrophages were quantified. Unexpectedly, IL-6 concentrations were significantly higher in culture supernatants from SOCS2^KI^ BMMs than those from WT BMMs after 30 nM of FSL-1 stimulation in the presence of increasing concentrations of IFN-γ and IL-10 (from 3 to 30 ng/ml) (**Figure 5A**). SOCS2^KI^ BMMs also produced higher levels of TNF-α in the IL-10 conditioning medium, even at the lowest FSL-1 concentration (3 nM) (**Figure 5B**). By contrast, phagocytosis was not significantly different between the two BMMs types, whatever the cytokines GM-CSF, IL-10, or IFN-γ used to pre-sensitize the BMMs (**Supplementary Figure 6**). In a context with the secretion of various cytokines, SOCS2 may have several effects on the cell responsiveness to MAMPS that are difficult to predict in culture.

### The SOCS2 LOF mutation increases the inflammatory response to *S. aureus* and worsens the infection outcome

**Figure 6:**
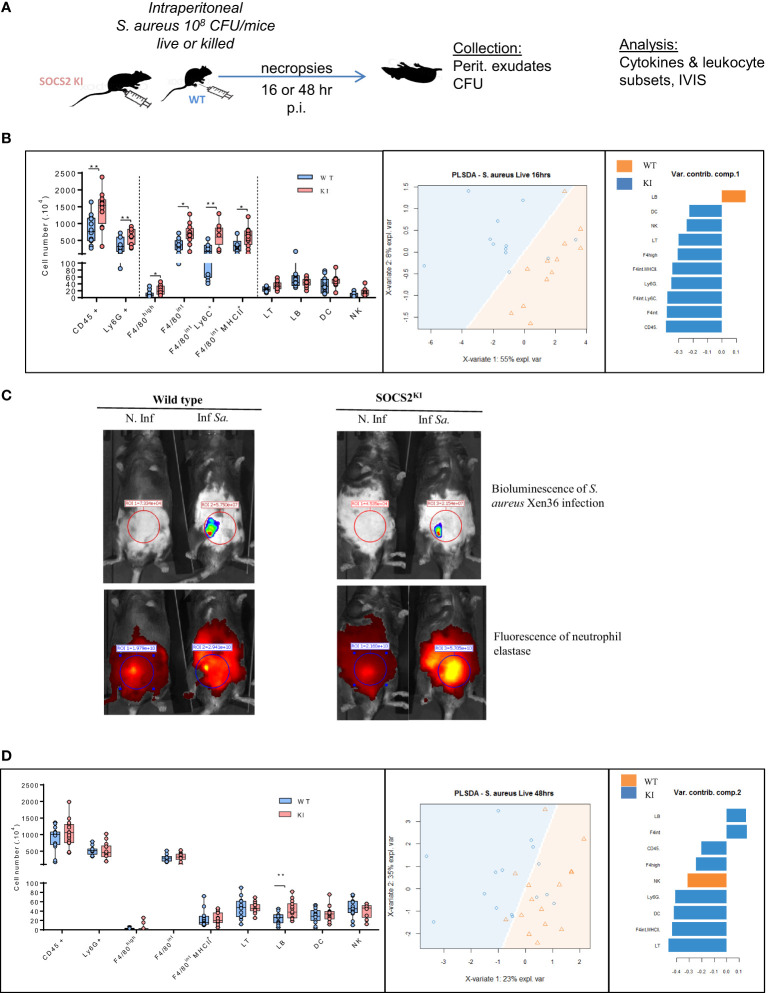
Immune cell analysis after 16 or 48 h of *S. aureus* peritonitis in SOCS2^KI^ or WT mice. **(A)** Experimental design: male SOCS2^KI^ or WT mice were peritoneally challenged with the *S. aureus* Xen36 strain at 10^8^ CFU/mice (N=12). **(B)** Immune cell composition in the peritoneal cavity analyzed by flow cytometry 16h after infection. PSLDA shows the hierarchical clustering of individual mice as a function of immune cell composition and the respective contribution of the quantitative variables to dimensions 1 or 2. (N=12) **(C)** *Ex-vivo* imaging of luminescence (*S. aureus*) and fluorescence intensity of elastase (neutrophil) 16h post-infection. Data are representative of two independent experiments. **(D)** Immune cell composition 48h post-infection in the peritoneal cavity analyzed by flow cytometry and PSLDA clustering (N=12). *P<0.05, **P<0.01, by multiple group comparison of ANOVA.

**Figure 7:**
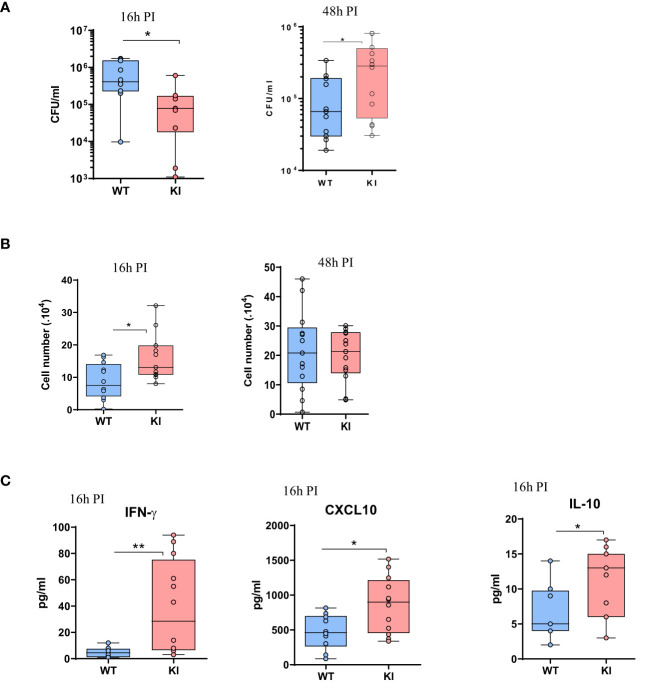
Analysis of bacterial fitness and the immune response after 16 or 48 h of *S. aureus* peritonitis in SOCS2^KI^ or WT mice. **(A)** Total live bacteria in the peritoneal lavage 16 and 48h post-infection. **(B)** Efferocytosis in the peritoneal cavity 16 and 48h post-infection (corresponding to total F4/80^+^Ly6G^+^ cells/live CD45^+^ cells) (39). **(C)** Cytokine concentrations (pg/mL) in the exudates at 16h post-infection determined by multiplexed ELISA. Results are represented by box and whisker plots showing the Min to Max (N=12). Statistical analysis was performed using the Mann-Whitney test and significant p values are indicated. *P<0.05, **P<0.01 vs. WT.

Peritoneal macrophages are central in the response to *Staphylococcus aureus* in a peritonitis model. To examine the impact of the LOF SOCS2 mutation, we inoculated SOCS2 KI and WT mice with a sublethal dose of *S. aureus*, as previously described (38) (**Figure 6A**). After 16 h of infection, peritoneal exudates were collected and the different cell types and cytokine concentrations were determined. The total cell number was higher in SOCS2^KI^ mice, due to a higher number of Ly6G^+^ neutrophils, and several macrophage subsets (defined as F4/80^int/+^ cells). A partial lest-square differential analysis (PLSDA) showed a clear dichotomy between the two genetic backgrounds using cell composition data (**Figure 6B**) indicating that the inflammatory response differed according to the SOCS2 variant. *In vivo* imaging showed higher neutrophil recruitment in SOCS2^KI^ than in WT mice, confirming the difference in inflammatory cell recruitment upon infection (**Figure 6C**). Interestingly, injection of heat-killed *S. aureus* (HKSA) failed to recapitulate the observed inflammatory response pattern (**Supplementary Figure 7**), suggesting that a dynamic response was established after inoculation of living bacteria, on the contrary to MAMPS injection. After 48 h of *S. aureus* infection, inflammatory cell recruitment was highly similar between the two types of mice (**Figure 6D**). As the cell numbers for neutrophils and macrophages were different at early stages of infection, we studied the bacterial fitness at 16, and 48 hours post-infection. In accordance with the increase in the number of neutrophils, there was a significantly lower number of live bacteria 16h post-infection (**Figure 7A**), associated with an increase in efferocytosis (as indicated by the increased number of F4/80^+^Ly6G^+^ cells) (**Figure 7B**). Interestingly, the peritoneal exudates of SOCS2^KI^ mice contained much more IFNγ and CXCL10 than those of WT mice (**Figure 7C**). By contrast, at 48h post-infection, the number of live bacteria was significantly higher in SOCS2^KI^ mice, indicating that, despite early recruitment of inflammatory cells, the infection was not contained in the SOCS2^KI^ mice and continued to progress.

## Discussion

Here, we provide evidence that the LOF R96C mutation of SOCS2 considerably alters the orchestration of the inflammatory response both *in vitro* and *in vivo*. Macrophages carrying the R96C SOCS2 variant showed heightened responsiveness to TLR ligands in terms of STAT activation and cytokine production, which characterize activated macrophages. Furthermore, *in vivo* studies using an *S. aureus* peritoneal infection model shows that R96C SOCS2 is associated with immune dysregulation, with elevated recruitment of several macrophage subsets and neutrophils at early stages and increased expression of pro-inflammatory cytokines and chemokines.

SOCS2 has two major functional domains: a central SH2 domain and a SOCS box. The SH2 domain directly interacts with JAK2, whereas the SOCS box mediates proteasomal degradation. Recently, Li et al. (2022) (11) showed that the R96C mutation in the SH2 domain of SOCS2 in mice does not compromise the domain integrity of the SOCS box, nor alter the ability of SOCS2 to recruit the SOCS box-associated-E3 ubiquitin ligase complex.

SOCS-2 is known to have an essential negative regulatory role in the growth hormone/IGF-I pathway (40). Here, we show that SOCS2^R96C^ KI mice display a similar increase in growth as previously reported for *Socs2^-/-^* mice, with an identical increase in body weight and bone length (40, 41). Moreover, our results are comparable to those of Metcalf and al, 2000 showing that adult SOCS2^-/-^ females attained the weight of WT male mice (40).

Overall, these observations confirm that the SOCS2^R96C^ KI mouse model provides a new tool to evaluate the functions of SOCS2 with a deficient SH2 domain. Indeed, this model, unlike the *Socs2* ^-/-^ model, makes it possible to study the interactions between proteins, in particular the contribution of SH2:pTyr binding to the function of SOCS2 in immune responses, while the expression of SOCS2 is maintained, lowering the risk of perturbing networks interaction amongst other SOCS proteins.

Furthermore, the inactivation of protein functions through the introduction of a point mutation by genome editing is nowadays a better option than knocking out the entire gene with the loss of protein expression, which has uncontrolled consequences like the possible deregulation of gene expression in the locus by large insertions, or disturbances of protein network, as for the SOCS-SOCS interactions.

Several studies have suggested that SOCS proteins participate in pattern recognition receptor (PRR) signaling. SOCS2 is a cytokine-inducible inhibitor of the JAK-STAT signaling pathway, with several studies reporting that SOCS2 is directly induced by TLR ligation in DCs and macrophages (14, 20). Hu et al. showed that various TLR ligands induce *Socs2* gene expression in human DCs (30). More precisely, TLR-4 signaling in monocyte-derived DCs induces the production of type I interferon, which in turn activates SOCS2 via STAT-3 and STAT-5. Moreover, Posselt et al. demonstrated that LPS stimulation of thioglycolate-induced mouse peritoneal macrophages results in significant upregulation of the mRNA levels of *Socs2*, which regulates IL-1beta and IL-10 (31). The same group showed that *Socs2* mRNA is induced in monocyte-derived DCs upon TLR-8 and NOD signaling, thus controlling the release of pro-inflammatory mediators from DCs (42). Our results do not confirm these findings. Indeed, BMM stimulation for 24 h with multiple TLR agonists failed to induce SOCS2 expression at the protein levels. This observation suggests the absence of any direct relationship between TLR-triggered pathways and SOCS2 expression. Rather, we believe that certain cytokines produced as a result of TLR stimulation may bind to their receptor in an autocrine fashion, and possibly induce SOCS2 expression, as we found that SOCS2^KI^ BMMs produce higher quantities of GM-CSF after FLS1 stimulation. Our results demonstrate that SOCS2 expression is not promoted directly by TLR-associated signaling pathways, and cytokines produced in the supernatant following TLR triggering were inadequate to do so (either by nature or amount).

Macrophages are key immune cells that play a crucial role in regulating and maintaining tissue homeostasis (43), as well as in immune defense against invading pathogens. Macrophages generated from bone-marrow progenitors or monocytes in the presence of GM-CSF are thought to be typically M1-like cells, producing pro-inflammatory cytokines upon stimulation with TLR ligands (44). The binding of GM-CSF to its receptor induces STAT5 phosphorylation, which in turn induces SOCS2 that cross-regulates back STAT5 activities (Review in 40,41). Consistent with this observation, we found that only GM-CSF, in contrast to the cytokines IFN-γ and IL-10, induced STAT5 phosphorylation and SOCS2 expression in BMMs from both WT and SOCS2^KI^ mice. Moreover, we found that the R96C mutation resulted in sustained STAT5 phosphorylation after GM-CSF stimulation. Moreover, Zhan et al. (45) recently reported that the amount of GM-CSF is a key factor in determining its biological activity. We investigated whether SOCS2 could have a direct activity on TLR-induced cytokine secretion by conditioning BMMs with increasing amounts of GM-CSF, and further stimulating them with the TLR-2 agonist FSL-1. Our results showed higher pro-inflammatory IL-6 production by SOCS2^KI^ BMMs that was dependent on the GM-CSF and FSL-1 ligand concentrations. Interestingly, not all cytokines are increased and the heightened response is specific to some factors (IL-6 vs. TNF-α). It is well known that SOCS1 production is induced by the cytokine IFN-γ via STAT1 (34) and that of SOCS3 by IL-10 via STAT3 (review in 46). Our results show that increasing the IL-10 concentration provokes significantly stronger pro-inflammatory cytokine responses by SOCS2^KI^ BMMs after TLR-2 engagement than by WT BMMs. We obtained comparable results in IFN-γ-conditioning medium, although the cytokine response appeared to be dependent on the TLR-ligand used. Previous studies have also shown that *Socs2* expression is upregulated by IFN-γ in DCs in human melanoma (47). Such data suggest that SOCS2 could negatively regulate SOCS3 protein levels and thus be a positive modulator of SOCS3-inhibited cytokine signaling cascades.

Here, we elucidated the impact of the LOF-R96C SOCS2 in an experimental model of *S. aureus* peritonitis. SOCS2^KI^ mice showed early intense myeloid cell recruitment, with elevated numbers of neutrophils and inflammatory macrophages in the peritoneal cavity associated with a decrease in bacterial content. Moreover, *S. aureus* peritonitis in SOCS2^KI^ mice resulted in higher IFN-γ, CXCL10, and IL-10 levels than in WT mice. Interestingly, Leech et al., 2017 (48) showed that IL-10 could play opposing roles during *S. aureus* systemic and localized infections. In particular, during localized infection, IL-10 production can plays detrimental role by facilitating bacterial persistence. Consistent with this observation, *Socs2* ^-/-^ mice show uncontrolled Th1 cell-mediated responses to *Toxoplasma gondii*, leading to death, suggesting an increased inflammatory response (17). SOCS2^KI^ mice showed an increase in macrophage efferocytosis at the early stages of infection, without a modification of phagocytosis. On the contrary, in an acute arthritis model, *Socs2* ^-/-^ macrophages exhibited reduced efferocytosis, but only for large peritoneal macrophages (F4/80^high^) (49). These authors concluded that SOCS2^KI^ mice exhibit less apoptosis and efferocytosis than WT mice at the late adjuvant-induced arthritis phase (49). At the late stage of *S. aureus* infection, SOCS2^KI^ mice showed a higher bacterial load, associated with a higher number of B lymphocytes. Given that R96C mutation resulted in prolonged phosphorylation of STAT5, which regulates FOXP3^+^ Tregs (50), further investigation on change in T-reg population can allow to gain insights resolution phase after *S. aureus* infection in mutated mice.

### Data limitations and perspectives of the study

Our results bring evidence that SOCS2 R96C mutation leads to a dysregulation of the inflammatory response. Along with infection with *S. aureus*, the initiation of the inflammatory reaction was similar between the two SOCS2 variants. However, later steps were characterized by increased production of cytokines, sustained recruitment of inflammatory cells, and a limited ability to control bacterial burden in R96C SOCS2 animals. Nevertheless, while we did not precisely identify the molecular and cellular mechanisms causing the impaired resolution phase in mutants, we showed *in vitro* that the R96C mutation led to a long-lasting phosphorylation of STAT5, which very likely contributes to inflammatory cytokines secretion *in vivo*. Further investigations will be necessary to fully decipher this process.

## Conclusion

We bring substantial *in vitro* and *in vivo* evidence arguing that the inflammatory context (such as the pattern of cytokine secretion) is changed by the R96C SOCS2 mutation. Consequently, we believe that rather than the absolute cell number, the intrinsic activity of newly recruited inflammatory cells (particularly monocytes) is determinant to ultimately regulate the immune response. Taken together these elements strongly suggest that SOCS2 mutation induces a defect in the resolution phase of the inflammatory response, which is a crucial step to avoid tissue damage and bacterial outgrowth. These results suggest that the R96C SOCS2 mutation could perturb the recruitment of immune cells and the regulation of the production of various pro-inflammatory cytokines during a bacterial infection. To conclude, our results show that the SOCS2 protein plays an important role in the immune response by controlling inflammatory cytokine production and reducing cell infiltration at the early stage of infection.

## Data availability statement

The raw data supporting the conclusions of this article will be made available by the authors, without undue reservation.

## Ethics statement

The animal study was approved by Animal Experimentation APAFIS#22936-2019112515186332, following the Guide for the Care and Use of Laboratory Animals (National Research Council, 1996) and in compliance with European directive 2010/63/UE under the supervision of authorized investigators. The study was conducted in accordance with the local legislation and institutional requirements.

## Author contributions

LG: Conceptualization, Formal analysis, Methodology, Supervision, Validation, Writing – original draft, Writing – review & editing. BG: Formal analysis, Investigation, Methodology, Writing – original draft. CT: Formal analysis, Methodology, Writing – original draft. CH: Formal analysis, Investigation, Methodology, Software, Validation, Writing – review & editing. GT: Conceptualization, Methodology, Software, Validation, Writing – review & editing, Writing – original draft. GF: Conceptualization, Resources, Supervision, Validation, Writing – original draft.

## Supplementary material

Supplementary Figure 1 Flow cytometry gating strategy. Flow cytometry gating strategy for the labeling and analysis of immune-cell sub-populations in peritoneal exudates. Leukocytes (identified as CD45^+^), were divided among B cells (CD19^+^), T cells (CD3^+^), neutrophils (Ly6G^+^), NK cells (NKp46^+^), dendritic cells (CD11c^+^), and two sub-populations of macrophages: resident macrophages (F4/80^high^) and inflammatory macrophages (F4/80^int^). This inflammatory macrophages were further separated into CD11c^low^/MHCII^+^ cells and CD11c^low^ Ly6C^+^ cells.

Supplementary Figure 2 The ratio of cell number/weight of spleens from SOCS2^KI^ or WT mice. **(A)** Spleen weight of adult SOCS2KI or WT mice. **(B)** The ratio of the cell number/weight of spleens from two-month-old male SOCS2^KI^ or WT mice (N=8). Statistical analysis was performed using the multiple t-tests of ANOVA. *P<0.05 vs WT.

Supplementary Figure 3 Flow cytometry analysis of macrophage/dendritic cell subsets in the spleens from SOCS2^KI^ or WT mice. Flow cytometry analysis of macrophage/dendritic cell subsets in the spleens from two-month-old male SOCS2^KI^ or WT mice (N=8). Statistical analysis was performed using the multiple t-tests of ANOVA.

Supplementary Figure 4 Phenotypic analysis of M-CSF derived BMMs from SOCS2^KI^ or WT mice. Bone marrow progenitors from SOCS2^KI^ and WT mice were cultured with 10 ng/ml of M-CSF for seven days. Adherent cells were harvested and analyzed by flow cytometry for F4/80, CD115, CD11b, and CD11c expression. SOCS, suppressor of cytokine signaling; WT, wild type; KI, SOCS2^KI^ mice.

Supplementary Figure 5 GM-CSF concentrations in BMM supernatant after TLR stimulation. BMMs from SOCS2^KI^ or WT mice were stimulated for 24 h with 3 to 100 nM FSL1 or CRX ligands. GM-CSF concentrations were measured in cell supernatants (N=5). Statistical analysis was performed using the multiple t-tests of ANOVA. **P<0.01 vs. WT.

Supplementary Figure 6 Phagocytic response of SOCS2^KI^ and WT BMMs to the *S. aureus*-GFP strain. BMMs were first primed with either GM-CSF, IFN-γ, or IL-10 for 24 h and then infected with *S. aureus* HG001-GFP for 1 h at 37°C or 4°C as control. The amount of live KI and WT BMMs with internal (BT-) GFP+ engulfed bacteria is represented (N=7). Statistical analysis was performed using the multiple t-test of ANOVA and significant p values are indicated. *P<0.05.

Supplementary Figure 7 Analysis of the immune response after 16 h of killed *S. aureus* peritonitis in SOCS2^KI^ or WT mice. **(A)** Immune cell composition in the peritoneal cavity of SOCS2^KI^ or WT mice. **(B)** PSLDA and hierarchical clustering of individual mice as a function of immune cell composition. The respective contribution of the quantitative variables to dimension 1 and 2 was determined. **(C)** Cytokine concentrations of TNF-α and IL-6 (pg/mL) in exudates. **(D)** Total F4/80^+^ Ly6G^+^ cells/CD45^+^ cells in the peritoneal cavity (39). (N=12). Statistical analysis was performed using the t-test and multiple t-tests of ANOVA and significant p values are indicated. *P<0.05 vs. WT.

Supplementary Figure 8 IL-6 and TNF-α response of BMMs to HKSA in GM-CSF, IFN-γ and IL-10 conditioning medium. Heatmap representation of IL-6 **(A)** or TNF-α **(B)** concentrations in supernatants of BMMs from SOCS2^KI^ or WT mice (Z-score transformation) after culture with increasing concentrations of HKSA (3 to 100 nM) and GM-CSF, IFN-γ or IL-10 (3 to 30 ng/ml). Results represent the mean ± SD of 5-6 independent donors. *P<0.05, **P<0.01, ***P<0.001, ****P<0.0001 vs. WT, by multiple group comparison of ANOVA.

Supplementary Table 1 List of antibodies used for flow cytometry in the study.

Supplementary Table 2 List of antibodies used for western blot in the study.

## References

[1] La Manna S De Benedictis I Marasco D. Proteomimetics of natural regulators of JAK–STAT pathway: novel therapeutic perspectives. Front Mol Biosci. (2022) 8:792546. doi: 10.3389/fmolb.2021.792546 35047557 PMC8762217

[2] Krebs DL Hilton DJ. SOCS: physiological suppressors of cytokine signaling. J Cell Sci. (2000) 113:2813–9. doi: 10.1242/jcs.113.16.2813 10910765

[3] Palmer DC Restifo NP. Suppressors of cytokine signaling (SOCS) in T cell differentiation, maturation, and function. Trends Immunol. (2009) 30:592–602. doi: 10.1016/j.it.2009.09.009 19879803 PMC2787651

[4] Knosp CA Carroll HP Elliott J Saunders SP Nel HJ Amu S. SOCS2 regulates T helper type 2 differentiation and the generation of type 2 allergic responses. J Exp Med. (2011) 208:1523–31. doi: 10.1084/jem.20101167 PMC3135359 21646394

[5] La Manna S Lopez-Sanz L Bernal S Jimenez-Castilla L Prieto I Morelli G. Antioxidant effects of PS5, a peptidomimetic of suppressor of cytokine signaling 1, in experimental atherosclerosis. Antioxidants. (2020) 9:754. doi: 10.3390/antiox9080754 32824091 PMC7465353

[6] Seki Y Inoue H Nagata N Hayashi K Fukuyama S Matsumoto K. SOCS-3 regulates onset and maintenance of TH2-mediated allergic responses. Nat Med. (2003) 9:1047–54. doi: 10.1038/nm896 12847520

[7] Chen Z Laurence A Kanno Y Pacher-Zavisin M Zhu B-M Tato C. Selective regulatory function of Socs3 in the formation of IL-17-secreting T cells. Proc Natl Acad Sci. (2006) 103:8137–42. doi: 10.1073/pnas.0600666103 PMC1459629 16698929

[8] Tanaka K Ichiyama K Hashimoto M Yoshida H Takimoto T Takaesu G. Loss of suppressor of cytokine signaling 1 in helper T cells leads to defective th17 differentiation by enhancing antagonistic effects of IFN-γ on STAT3 and smads. J Immunol. (2008) 180:3746–56. doi: 10.4049/jimmunol.180.6.3746 18322180

[9] Knosp CA Schiering C Spence S Carroll HP Nel HJ Osbourn M. Regulation of foxp3 ^+^ Inducible regulatory T cell stability by SOCS2. J Immunol. (2013) 190:3235–45. doi: 10.4049/jimmunol.1201396 PMC3607399 23455506

[10] McBerry C Gonzalez RMS Shryock N Dias A Aliberti J. SOCS2-induced proteasome-dependent TRAF6 degradation: A common anti-inflammatory pathway for control of innate immune responses. PloS One. (2012) 7:e38384. doi: 10.1371/journal.pone.0038384 22693634 PMC3367914

[11] Li K Meza Guzman LG Whitehead L Leong E Kueh A Alexander WS. SOCS2 regulation of growth hormone signaling requires a canonical interaction with phosphotyrosine. Biosci Rep. (2022) 42:BSR20221683. doi: 10.1042/BSR20221683 36398696 PMC9742514

[12] Horvat S Medrano JF. Lack of socs2 expression causes the high-growth phenotype in mice. Genomics. (2001) 72:209–12. doi: 10.1006/geno.2000.6441 11401434

[13] Linossi EM Nicholson SE. The SOCS box-Adapting proteins for ubiquitination and proteasomal degradation. IUBMB Life. (2012) 64:316–23. doi: 10.1002/iub.1011 22362562

[14] Linossi EM Li K Veggiani G Tan C Dehkhoda F Hockings C. Discovery of an exosite on the SOCS2-SH2 domain that enhances SH2 binding to phosphorylated ligands. Nat Commun. (2021) 12:7032. doi: 10.1038/s41467-021-26983-5 34857742 PMC8640019

[15] Kim WS Kim MJ Kim DO Byun J-E Huy H Song HY. Suppressor of cytokine signaling 2 negatively regulates NK cell differentiation by inhibiting JAK2 activity. Sci Rep. (2017) 7:46153. doi: 10.1038/srep46153 28383049 PMC5382670

[16] Esper L Roman-Campos D Lara A Brant F Castro LL Barroso A. Role of SOCS2 in modulating heart damage and function in a murine model of acute chagas disease. Am J Pathol. (2012) 181:130–40. doi: 10.1016/j.ajpath.2012.03.042 PMC3388166 22658486

[17] MaChado FS Johndrow JE Esper L Dias A Bafica A Serhan CN. Anti-inflammatory actions of lipoxin A4 and aspirin-triggered lipoxin are SOCS-2 dependent. Nat Med. (2006) 12:330–4. doi: 10.1038/nm1355 16415877

[18] Brant F Miranda AS Esper L Gualdrón-López M Cisalpino D de Souza D da G. Suppressor of cytokine signaling 2 modulates the immune response profile and development of experimental cerebral malaria. Brain Behav Immun. (2016) 54:73–85. doi: 10.1016/j.bbi.2016.01.002 26765997

[19] Rupp R Senin P Sarry J Allain C Tasca C Ligat L. A point mutation in suppressor of cytokine signalling 2 (Socs2) increases the susceptibility to inflammation of the mammary gland while associated with higher body weight and size and higher milk production in a sheep model. PloS Genet. (2015) 11:e1005629. doi: 10.1371/journal.pgen.1005629 26658352 PMC4676722

[20] Oget C Teissier M Astruc J-M Tosser-Klopp G Rupp R. Alternative methods improve the accuracy of genomic prediction using information from a causal point mutation in a dairy sheep model. BMC Genomics. (2019) 20:719. doi: 10.1186/s12864-019-6068-4 31533617 PMC6751880

[21] Duncan SA Baganizi DR Sahu R Singh SR Dennis VA. SOCS proteins as regulators of inflammatory responses induced by bacterial infections: A review. Front Microbiol. (2017) 8:2431. doi: 10.3389/fmicb.2017.02431 29312162 PMC5733031

[22] Tannahill GM Elliott J Barry AC Hibbert L Cacalano NA Johnston JA. SOCS2 can enhance interleukin-2 (IL-2) and IL-3 signaling by accelerating SOCS3 degradation. Mol Cell Biol. (2005) 25:9115–26. doi: 10.1128/MCB.25.20.9115-9126.2005 PMC1265772 16199887

[23] Hu J Winqvist O Flores-Morales A Wikström A-C Norstedt G. SOCS2 influences LPS induced human monocyte-derived dendritic cell maturation. PloS One. (2009) 4:e7178. doi: 10.1371/journal.pone.0007178 19779605 PMC2744869

[24] Carow B Rottenberg ME. SOCS3, a major regulator of infection and inflammation. Front Immunol. (2014) 5:58. doi: 10.3389/fimmu.2014.00058 24600449 PMC3928676

[25] Pelletier S Gingras S Green DR. Mouse genome engineering via CRISPR-cas9 for study of immune function. Immunity. (2015) 42:18–27. doi: 10.1016/j.immuni.2015.01.004 25607456 PMC4720985

[26] Herbert S Ziebandt A-K Ohlsen K Schäfer T Hecker M Albrecht D. Repair of global regulators in *staphylococcus aureus* 8325 and comparative analysis with other clinical isolates. Infect Immun. (2010) 78:2877–89. doi: 10.1128/IAI.00088-10 PMC2876537 20212089

[27] Rohart F Gautier B Singh A Lê Cao K-A. mixOmics: An R package for ‘omics feature selection and multiple data integration. PloS Comput Biol. (2017) 13:e1005752. doi: 10.1371/journal.pcbi.1005752 29099853 PMC5687754

[28] Cunnick J Kaur P Cho Y Groffen J Heisterkamp N. Use of bone marrow-derived macrophages to model murine innate immune responses. J Immunol Methods. (2006) 311:96–105. doi: 10.1016/j.jim.2006.01.017 16563426

[29] Dalpke A Heeg K Bartz H Baetz A. Regulation of innate immunity by suppressor of cytokine signaling (SOCS) proteins. Immunobiology. (2008) 213:225–35. doi: 10.1016/j.imbio.2007.10.008 18406369

[30] Hu J Lou D Carow B Winerdal ME Rottenberg M Wikström A-C. LPS regulates SOCS2 transcription in a type I interferon dependent autocrine-paracrine loop. PloS One. (2012) 7:e30166. doi: 10.1371/journal.pone.0030166 22291912 PMC3264591

[31] Posselt G Schwarz H Duschl A Horejs-Hoeck J. Suppressor of cytokine signaling 2 is a feedback inhibitor of TLR-induced activation in human monocyte-derived dendritic cells. J Immunol. (2011) 187:2875–84. doi: 10.4049/jimmunol.1003348 21844389

[32] Vitali C Bassani C Chiodoni C Fellini E Guarnotta C Miotti S. SOCS2 controls proliferation and stemness of hematopoietic cells under stress conditions and its deregulation marks unfavorable acute leukemias. Cancer Res. (2015) 75:2387–99. doi: 10.1158/0008-5472.CAN-14-3625 25858143

[33] Rico-Bautista E Flores-Morales A Fernández-Pérez L. Suppressor of cytokine signaling (SOCS) 2, a protein with multiple functions. Cytokine Growth Factor Rev. (2006) 17:431–9. doi: 10.1016/j.cytogfr.2006.09.008 17070092

[34] Liu H Wang W Liu C. Increased expression of IFN−γ in preeclampsia impairs human trophoblast invasion via a SOCS1/JAK/STAT1 feedback loop. Exp Ther Med. (2020) 21:112. doi: 10.3892/etm.2020.9544 33335575 PMC7739872

[35] Gao Y Zhao H Wang P Wang J Zou L. The roles of SOCS3 and STAT3 in bacterial infection and inflammatory diseases. Scand J Immunol. (2018) 88:e12727. doi: 10.1111/sji.12727 30341772

[36] Brady NJ Farrar MA Schwertfeger KL. STAT5 deletion in macrophages alters ductal elongation and branching during mammary gland development. Dev Biol. (2017) 428:232–44. doi: 10.1016/j.ydbio.2017.06.007 PMC5621646 28606561

[37] Sobah ML Liongue C Ward AC. SOCS proteins in immunity, inflammatory diseases, and immune-related cancer. Front Med. (2021) 8:727987. doi: 10.3389/fmed.2021.727987 PMC8481645 34604264

[38] Kim HK Missiakas D Schneewind O. Mouse models for infectious diseases caused by Staphylococcus aureus. J Immunol Methods. (2014) 410:88–99. doi: 10.1016/j.jim.2014.04.007 24769066 PMC6211302

[39] Meriwether D Jones AE Ashby JW Solorzano-Vargas RS Dorreh N Noori S. Macrophage COX2 mediates efferocytosis, resolution reprogramming, and intestinal epithelial repair. Cell Mol Gastroenterol Hepatol. (2022) 13:1095–120. doi: 10.1016/j.jcmgh.2022.01.002 PMC8873959 35017061

[40] Metcalf D Greenhalgh CJ Viney E Willson TA Starr R Nicola NA. Gigantism in mice lacking suppressor of cytokine signalling-2. Nature. (2000) 405:1069–73. doi: 10.1038/35016611 10890450

[41] Greenhalgh CJ Miller ME Hilton DJ Lund PK. Suppressors of cytokine signaling: Relevance to gastrointestinal function and disease. Gastroenterology. (2002) 123:2064–81. doi: 10.1053/gast.2002.37068 12454862

[42] Schwarz H Posselt G Wurm P Ulbing M Duschl A Horejs-Hoeck J. TLR8 and NOD signaling synergistically induce the production of IL-1β and IL-23 in monocyte-derived DCs and enhance the expression of the feedback inhibitor SOCS2. Immunobiology. (2013) 218:533–42. doi: 10.1016/j.imbio.2012.06.007 22795647

[43] Lavin Y Mortha A Rahman A Merad M. Regulation of macrophage development and function in peripheral tissues. Nat Rev Immunol. (2015) 15:731–44. doi: 10.1038/nri3920 PMC4706379 26603899

[44] Murray PJ Allen JE Biswas SK Fisher EA Gilroy DW Goerdt S. Macrophage activation and polarization: nomenclature and experimental guidelines. Immunity. (2014) 41:14–20. doi: 10.1016/j.immuni.2014.06.008 25035950 PMC4123412

[45] Zhan Y Lew AM Chopin M. The pleiotropic effects of the GM-CSF rheostat on myeloid cell differentiation and function: more than a numbers game. Front Immunol. (2019) 10:2679. doi: 10.3389/fimmu.2019.02679 31803190 PMC6873328

[46] Letellier E Haan S. SOCS2: physiological and pathological functions. Front Biosci Elite Ed. (2016) 8:189–204. doi: 10.2741/E760 26709655

[47] Nirschl CJ Suárez-Fariñas M Izar B Prakadan S Dannenfelser R Tirosh I. IFNγ-dependent tissue-immune homeostasis is co-opted in the tumor microenvironment. Cell. (2017) 170:127–141.e15. doi: 10.1016/j.cell.2017.06.016 28666115 PMC5569303

[48] Leech JM Lacey KA Mulcahy ME Medina E McLoughlin RM. IL-10 Plays Opposing Roles during *Staphylococcus aureus* Systemic and Localized Infections. J Immunol. (2017) 198:2352–65. doi: 10.4049/jimmunol.1601018 PMC5337812 28167629

[49] Cramer A Galvão I Venturini de Sá N Gaio P Fernanda de Melo Oliveira N Rates Gonzaga Santos N. Role of Suppressor of cytokine signaling 2 during the development and resolution of an experimental arthritis. Cell Immunol. (2022) 372:104476. doi: 10.1016/j.cellimm.2021.104476 35033752

[50] Jones DM Read KA Oestreich KJ. Dynamic roles for IL-2–STAT5 signaling in effector and regulatory CD4+ T cell populations. J Immunol. (2020) 205:1721–30. doi: 10.4049/jimmunol.2000612 PMC7513451 32958706

