# Supplementary figures and images for: A loss of function mutation in SOCS2 results in increased inflammatory response of macrophages to TLR ligands and Staphylococcus aureus
Source: Front Immunol. 2024 Aug 9;15:1397330. doi: 10.3389/fimmu.2024.1397330 (PMC11341364; doi:10.3389/fimmu.2024.1397330)

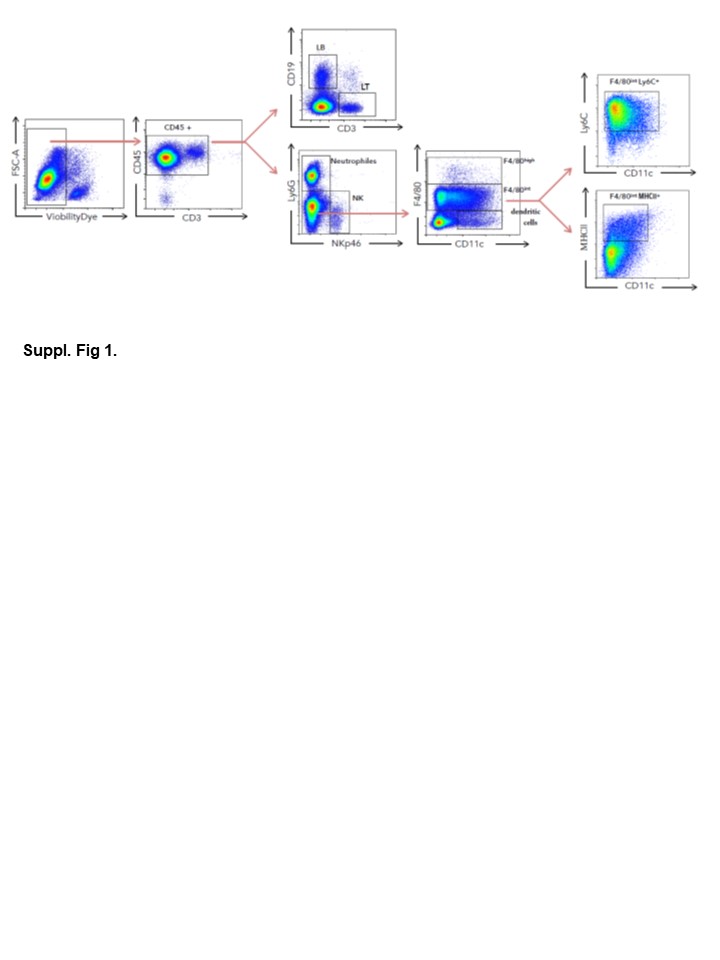

Supplement: Supplementary Figure 1 — Flow cytometry gating strategy. Flow cytometry gating strategy for the labeling and analysis of immune-cell sub-populations in peritoneal exudates. Leukocytes (identified as CD45+), were divided among B cells (CD19+), T cells (CD3+), neutrophils (Ly6G+), NK cells (NKp46+), dendritic cells (CD11c+), and two sub-populations of macrophages: resident macrophages (F4/80high) and inflammatory macrophages (F4/80int). This inflammatory macrophages were further separated into CD11clow/MHCII+ cells and CD11clow Ly6C+ cells. [file Image_1.jpeg]

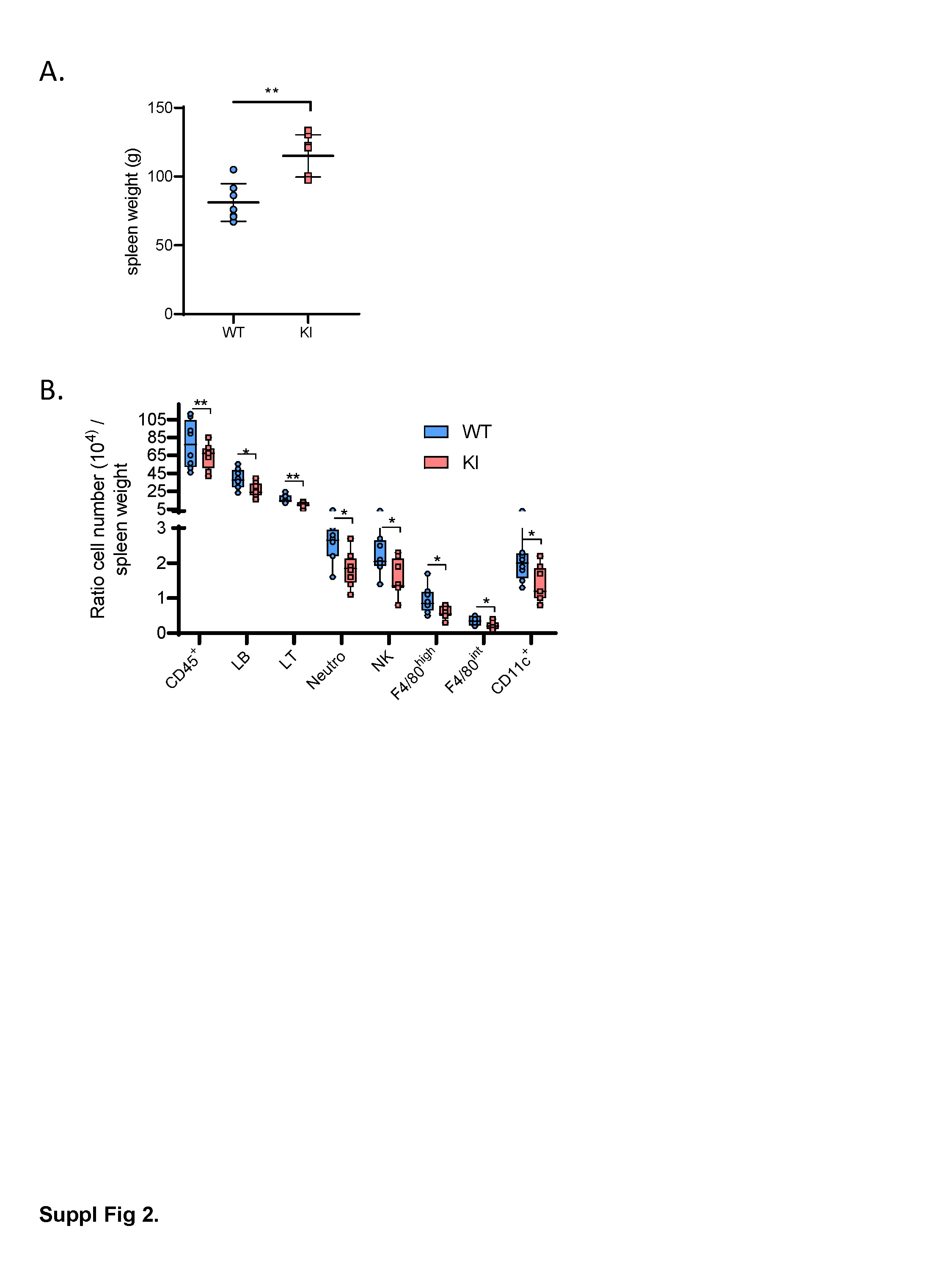

Supplement: Supplementary Figure 2 — The ratio of cell number/weight of spleens from SOCS2KI or WT mice. (A) Spleen weight of adult SOCS2KI or WT mice. (B) The ratio of the cell number/weight of spleens from two-month-old male SOCS2KI or WT mice (N=8). Statistical analysis was performed using the multiple t-tests of ANOVA. *P<0.05 vs WT. [file Image_2.jpeg]

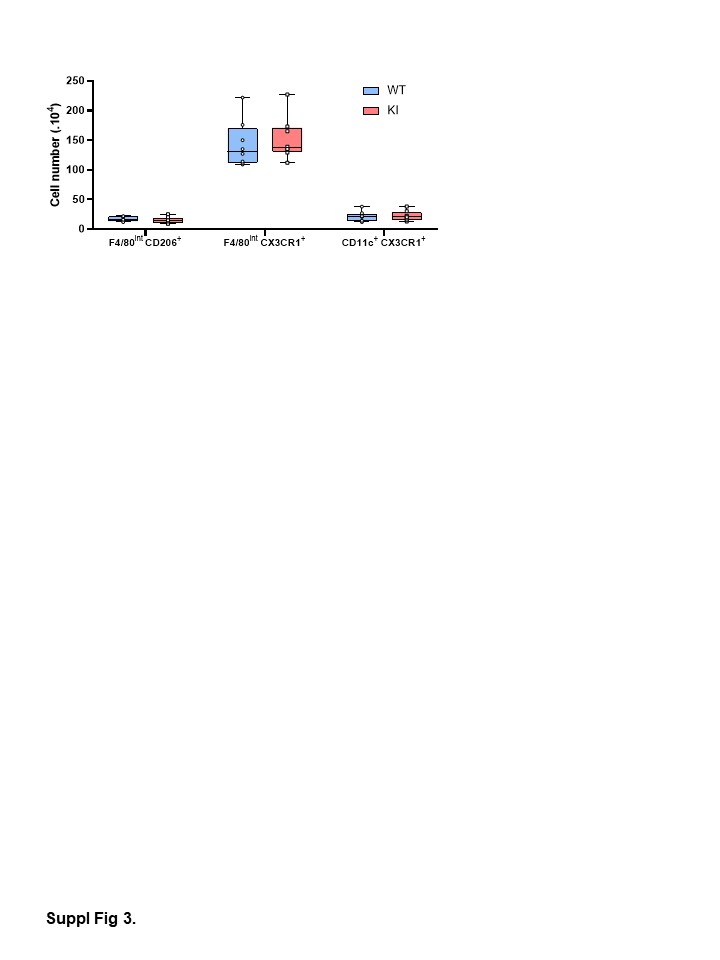

Supplement: Supplementary Figure 3 — Flow cytometry analysis of macrophage/dendritic cell subsets in the spleens from SOCS2KI or WT mice. Flow cytometry analysis of macrophage/dendritic cell subsets in the spleens from two-month-old male SOCS2KI or WT mice (N=8). Statistical analysis was performed using the multiple t-tests of ANOVA. [file Image_3.jpeg]

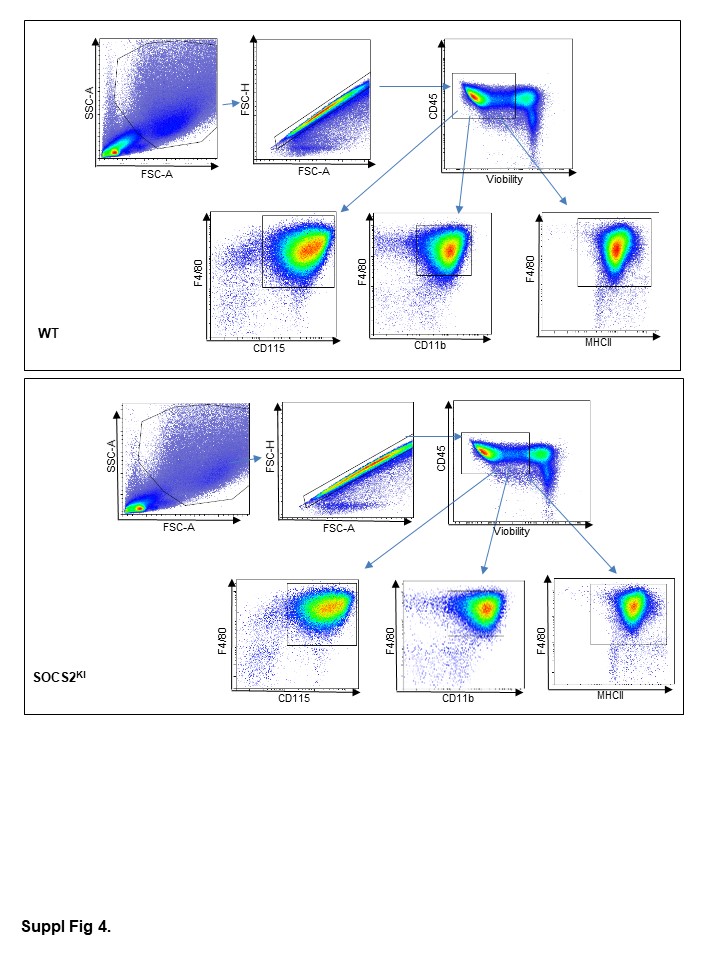

Supplement: Supplementary Figure 4 — Phenotypic analysis of M-CSF derived BMMs from SOCS2KI or WT mice. Bone marrow progenitors from SOCS2KI and WT mice were cultured with 10 ng/ml of M-CSF for seven days. Adherent cells were harvested and analyzed by flow cytometry for F4/80, CD115, CD11b, and CD11c expression. SOCS, suppressor of cytokine signaling; WT, wild type; KI, SOCS2KI mice. [file Image_4.jpeg]

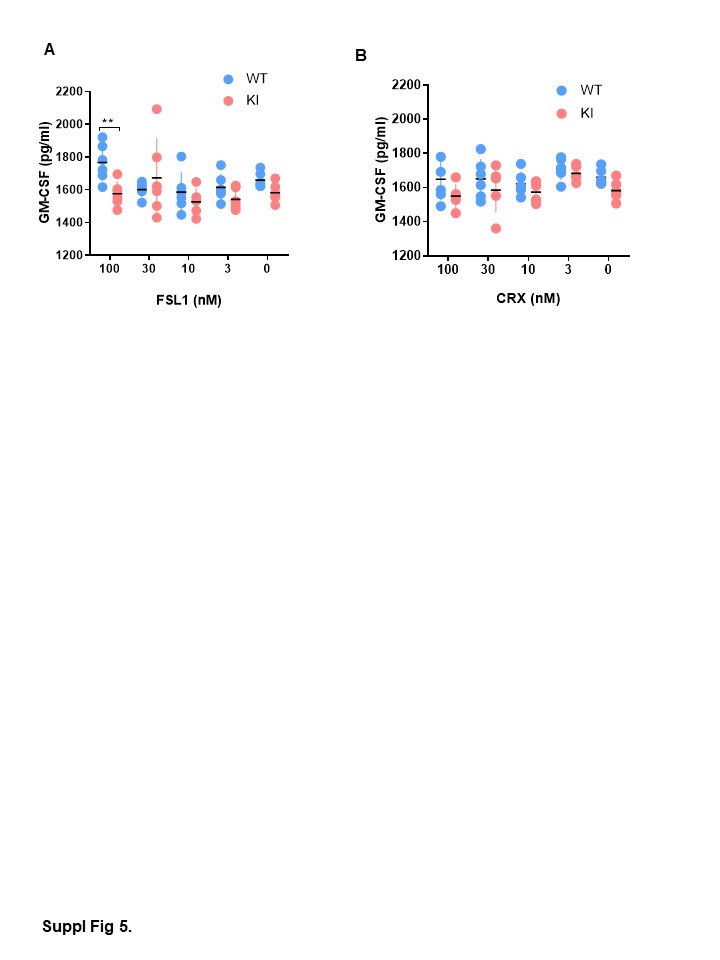

Supplement: Supplementary Figure 5 — GM-CSF concentrations in BMM supernatant after TLR stimulation. BMMs from SOCS2KI or WT mice were stimulated for 24 h with 3 to 100 nM FSL1 or CRX ligands. GM-CSF concentrations were measured in cell supernatants (N=5). Statistical analysis was performed using the multiple t-tests of ANOVA. **P<0.01 vs. WT. [file Image_5.jpeg]

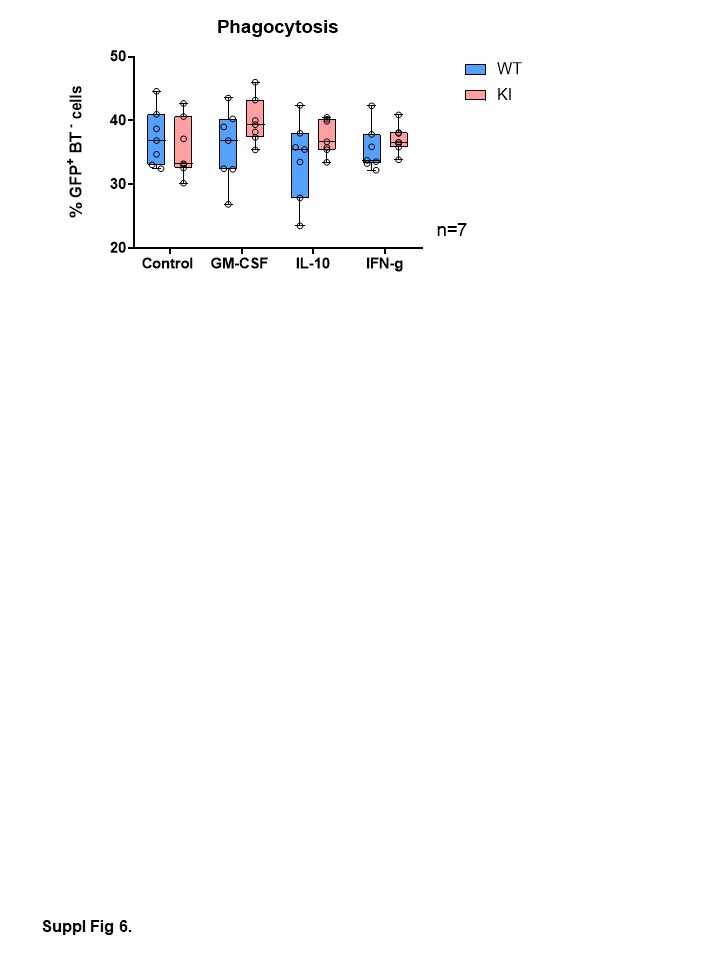

Supplement: Supplementary Figure 6 — Phagocytic response of SOCS2KI and WT BMMs to the S. aureus-GFP strain. BMMs were first primed with either GM-CSF, IFN-γ, or IL-10 for 24 h and then infected with S. aureus HG001-GFP for 1 h at 37°C or 4°C as control. The amount of live KI and WT BMMs with internal (BT-) GFP+ engulfed bacteria is represented (N=7). Statistical analysis was performed using the multiple t-test of ANOVA and significant p values are indicated. *P<0.05. [file Image_6.jpeg]

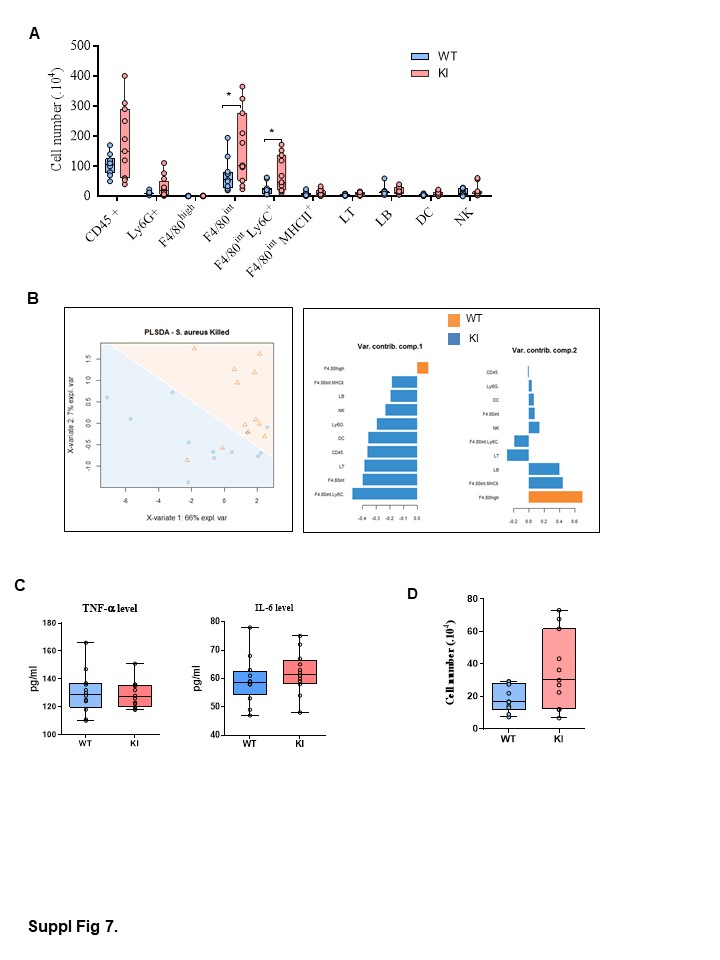

Supplement: Supplementary Figure 7 — Analysis of the immune response after 16 h of killed S. aureus peritonitis in SOCS2KI or WT mice. (A) Immune cell composition in the peritoneal cavity of SOCS2KI or WT mice. (B) PSLDA and hierarchical clustering of individual mice as a function of immune cell composition. The respective contribution of the quantitative variables to dimension 1 and 2 was determined. (C) Cytokine concentrations of TNF-α and IL-6 (pg/mL) in exudates. (D) Total F4/80+ Ly6G+ cells/CD45+ cells in the peritoneal cavity (39). (N=12). Statistical analysis was performed using the t-test and multiple t-tests of ANOVA and significant p values are indicated. *P<0.05 vs. WT. [file Image_7.jpeg]

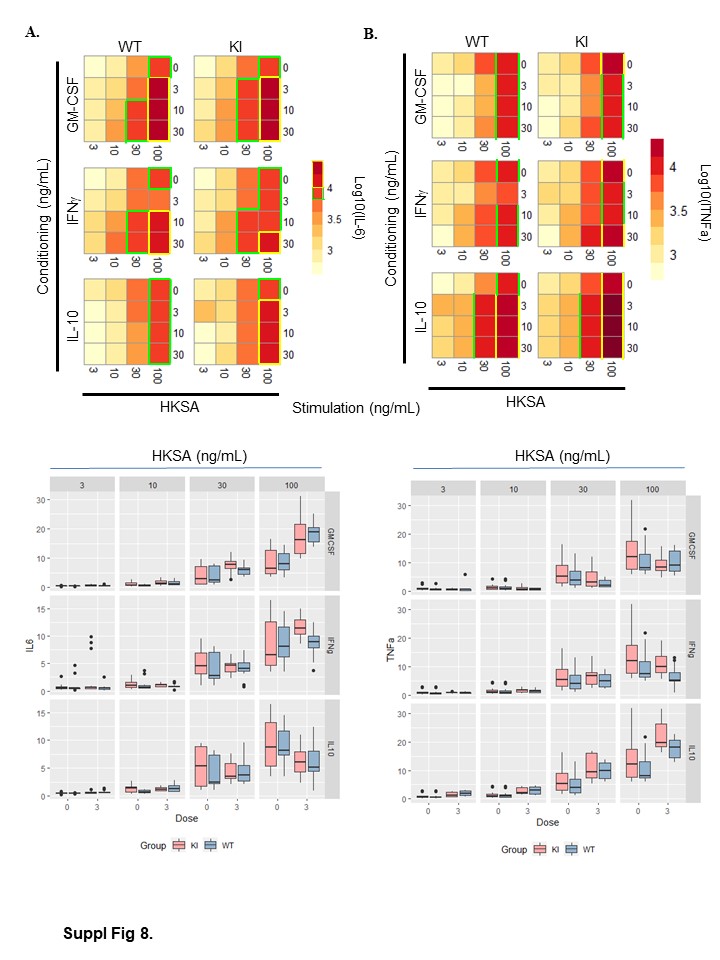

Supplement: Supplementary Figure 8 — IL-6 and TNF-α response of BMMs to HKSA in GM-CSF, IFN-γ and IL-10 conditioning medium. Heatmap representation of IL-6 (A) or TNF-α (B) concentrations in supernatants of BMMs from SOCS2KI or WT mice (Z-score transformation) after culture with increasing concentrations of HKSA (3 to 100 nM) and GM-CSF, IFN-γ or IL-10 (3 to 30 ng/ml). Results represent the mean ± SD of 5-6 independent donors. *P<0.05, **P<0.01, ***P<0.001, ****P<0.0001 vs. WT, by multiple group comparison of ANOVA. [file Image_8.jpeg]
