# Supplementary material for: A loss of function mutation in SOCS2 results in increased inflammatory response of macrophages to TLR ligands and Staphylococcus aureus
Source: Front Immunol. 2024 Aug 9;15:1397330. doi: 10.3389/fimmu.2024.1397330 (PMC11341364; doi:10.3389/fimmu.2024.1397330)
Supplement: Supplementary Table 1 — List of antibodies used for flow cytometry in the study. [file Table_1.pdf]

Antigen	Fluorochrome	Clone	source
Ly-6G	VioBlue	REAfinity™, clone REA526	Miltenyi Biotec
CD45	VioGreen	REAfinity™, clone REA737	Miltenyi Biotec
CD335 (NKp46)	PE	REAfinity™, clone REA815	Miltenyi Biotec
CD19	PE-Vio 615	REAfinity™, clone REA749	Miltenyi Biotec
CD11c	PE-Vio 770	REAfinity™, clone REA754	Miltenyi Biotec
F4/80	APC	REAfinity™, clone REA126	Miltenyi Biotec
CD3	APC-Vio770	REAfinity™, clone REA641	Miltenyi Biotec
Ly-6C	VioBlue	REAfinity™, clone REA796	Miltenyi Biotec
Ly-6G	FITC	REAfinity™, clone REA526	Miltenyi Biotec
CD335 (NKp46)	FITC	REAfinity™, clone REA815	Miltenyi Biotec
CD19	FITC	REAfinity™, clone REA749	Miltenyi Biotec
CD3	FITC	REAfinity™, clone REA641	Miltenyi Biotec
CD206 (MMR)	PE	Rat, C068C2	Biolegend
CX3CR1	PerCP/Cyanine5.5	SA011F11	Biolegend
CD64	APC-Vio770	REAfinity™, clone REA286	Miltenyi Biotec
CD11b	APC-Vio770	REAfinity™, clone REA592	Miltenyi Biotec
MHCII	VioBlue	REAfinity™, clone REA813	Miltenyi Biotec

Table 1.
