# Supplementary material for: A loss of function mutation in SOCS2 results in increased inflammatory response of macrophages to TLR ligands and Staphylococcus aureus
Source: Front Immunol. 2024 Aug 9;15:1397330. doi: 10.3389/fimmu.2024.1397330 (PMC11341364; doi:10.3389/fimmu.2024.1397330)
Supplement: Supplementary Table 2 — List of antibodies used for western blot in the study. [file Table_2.pdf]

Antigen	Clone	Reference	Source	Specificity
SOCS2	Polyclonal	2779	Cell Signalling	SOCS2 Antibody detects endogenous levels of SOCS2 protein.
STAT5	D206Y	94205	Cell Signalling	Rabbit mAb recognizes endogenous levels of total Stat5 protein. This antibody recognizes both alpha and beta isoforms of Stat5 (Stat5a and Stat5b)
phospho STAT5 (Tyr 694)	C11C5	9359	Cell Signalling	Rabbit mAb detects endogenous levels of Stat5a only when phosphorylated at Tyr694 and Stat5b when phosphorylated at Tyr699

Table 2.
